# Understanding feeding practices of Latinx mothers of infants and toddlers at risk for childhood obesity: A qualitative study

**DOI:** 10.1111/mcn.12983

**Published:** 2020-03-05

**Authors:** Hannah McFarren, Christian Vazquez, Elizabeth A. Jacobs, Giovanna Dela Tejera, Megan Gray

**Affiliations:** ^1^ Dell Medical School The University of Texas at Austin Austin Texas; ^2^ Steve Hicks School of Social Work The University of Texas at Austin Austin Texas; ^3^ College of Natural Sciences The University of Texas at Austin Austin Texas

**Keywords:** child feeding, childhood obesity, infant and child nutrition, infant feeding, Latinx health, minority health, parentingqualitative methods

## Abstract

Infancy and toddlerhood are critical stages for the development of habits that can lead to future obesity, and caregivers have an important influence on these habits. We conducted this qualitative semistructured interview study to explore the feeding practices of Latinx mothers of young children who are at risk for childhood obesity in order to identify targets for obesity prevention. We interviewed Latinx mothers (*N* = 14) of a child ages 6–18 months with a weight‐for‐length ratio > 85th percentile at the time of recruitment. Two researchers independently read through the interviews, identified sections of the interviews pertaining to feeding, and used constant comparative methods to identify the following common themes: mothers overwhelmingly reported permissive feeding styles, driving overfeeding and frequent night‐time feeding. Mothers expressed some difficulty with transitioning to solid foods and reported desiring to feed their child healthy foods by minimizing juice and giving vegetables. Paediatricians and WIC staff were viewed by mothers as trustworthy sources of nutrition information. Most identified a connection between their child's weight and diet, but many lacked the insight or capacity to change their current practices. The mothers in our study provide insight into factors that may predispose young children to obesity and thus potential avenues to support these families. Healthcare providers can better serve them by giving clear, actionable advice on healthy feeding practices for their child, while understanding constraints that may make healthy habits difficult to implement. Paediatricians should be honest about their child's weight status early on to allow time for intervention.

**Key messages**

- There are several potential obesity prevention intervention targets regarding early childhood feeding including timing and quantity of meals. Healthcare delivery methods and models should be designed to address these target for intervention to help mothers establish healthier feeding practices.
- In this study, mothers tended to practice permissive feeding styles, driving overfeeding. Many mothers did not perceive their child's weight as an issue of concern for a number of reasons.
- Further research is needed to investigate the relationship between feeding styles and childhood obesity as well as the impact of nutrition interventions on feeding style.

## 1 INTRODUCTION

Childhood obesity is a significant public health concern. It is estimated that 16.9% of children ages 2 to 4 years in Texas and 13.9% of children ages 2 to 5 years in the United States are obese (Hales, Fryar, Carroll, Freedman, & Ogden, 2018; Pan et al., 2016). Many children who are obese during early childhood will continue to be obese into adulthood, placing them at risk for type two diabetes, cardiovascular disease, osteoarthritis, hypertension, and chronic kidney disease, among other health issues (Nemiary, Shim, Mattox, & Holden, 2012).

Racial and ethnic minorities experience large disparities in childhood obesity rates. Latinx children are two times more likely than black children and three times more likely than white or Asian children to be obese (Ogden et al., 2016). Additionally, Hispanic children are now more than one fourth of the population under 1 year of age in the United States (Gaffney et al., 2018). Prior obesity research has primarily focused on understanding, preventing, and treating childhood obesity among older children and youth, and obesity studies regarding infants have focused mostly on breastfeeding versus formula feeding. However, a growing body of evidence shows that risk factors for the development of childhood obesity begin at the prenatal, infant, and toddler stages (Weng, Redsell, Swift, Yang, & Glazebrook, 2012; Geserick et al., 2018). In a systematic review of studies assessing rapid weight gain in the first 2 years of life, 45 out of 46 studies found an association between rapid weight gain in infancy and subsequent overweight status in childhood (Woo Baidal et al., 2016).

Additionally, evidence suggests that eating habits are established as young as 2 years of age, and, like weight, have been shown to track into adulthood (Harrison, Brodribb, & Hepworth, 2017). Meal choices, timing and quantity of feeding, feeding styles, and perception of healthy child weight have been identified as key factors for the development of childhood obesity (Gross, Mendelsohn, Fierman, Racine, & Messito, 2012; Harrison et al., 2017; Li, Scanlon, May, Rose, & Birch, 2014; Azad et al., 2018). Focusing on early childhood may help to get ahead of childhood obesity by setting a firm foundation for the child and the parent to learn and establish healthy habits (Helle, Hillesund, Wills, & Øverby, 2019).

Previous investigators have suggested that parental feeding styles, or the structure and responsiveness of the parent to the child's hunger and satiety cues (Chaidez & Kaiser, 2011), shape the early feeding environment (Horodynski et al., 2018). Among the four parenting styles described in Baumrind's theory of feeding styles, an authoritative feeding style is associated with a healthier dietary pattern (more vegetables and dairy), compared to indulgent, uninvolved, and authoritarian styles (Chaidez & Kaiser, 2011). However, Hispanic parents are more likely to practice indulgent feeding styles (Chaidez & Kaiser, 2011), which are correlated with higher body mass index in longitudinal studies (Shloim, Edelson, Martin, & Hetherington, 2015). These feeding styles are also associated with feeding to soothe, and it has been shown that children who do not learn to self soothe may also continue to cry throughout the night (McGeorge, Milne, Cotton, & Whelan, 2015), establishing a cycle of night‐time feeding. Some studies also suggest that Latinx families have different standards and ideals for what constitutes a healthy child (Isasi, Rastogi, & Molina, 2016) and may consider higher infant weight a “safety net” and a sign of good mothering (Harrison et al., 2017).

To our knowledge, there are no studies to date focused on feeding practices among Latinx mothers of children under 2 years of age who are at risk for childhood obesity in the southern United States region. The purpose of this research was to explore parenting practices and experiences generally. However, this particular study focuses on feeding practices and explores how practices affect and are affected by soothing practices, the child's sleep patterns, routines, and social stressors among Latinx mothers of children at risk for childhood obesity. In doing so, we hope to identify potential targets for successful, culturally sensitive obesity prevention counselling and programming.

## 2 METHODS

### 2.1 Recruitment and eligibility

Parents were eligible to participate in interviews regarding parenting practices and experiences if they had a child aged 6 to 18 months with a weight‐for‐length ratio greater than or equal to the 85th percentile within the past 3 months at time of recruitment, spoke English or Spanish, and if the parent/carer age was over 18 years. A nonbiological parent was included as part of the sample. See Table 1 below for a description of the participants' demographic data. Participants were recruited at a Federally Qualified Health Center in Central Texas. This health centre primarily serves Latinx patients on Medicaid, Children's Health Insurance Program, and local insurance plans. A list of potential participants was created ahead of time based on these criteria by searching the health record for upcoming check‐up appointments, at which the child's growth is measured. The researcher went to the clinic on the days of potential participant appointments and informed the paediatricians of which patients were eligible. Caregivers were recruited in person with the permission of the paediatrician after the paediatrician had already discussed the child's weight status with the caregiver. Informed consent was obtained, and the interview was scheduled at a time and location that was convenient for the caregiver. A $10 grocery store gift card was given both at the time of recruitment and after completion of the interview.

**Table 1:** Demographics

Mothers	
Total number	*n* = 14
Ethnicity	
Hispanic	*n* = 14
Language of preference	
Spanish	*n* = 11
English	*n* = 3
Age of mother	
Age 22–26	*n* = 5
Age 27–33	*n* = 4
Age 34–41	*n* = 5
Median Age	30 years
Relationship to child	
Biological mother	*n* = 13
Foster mother	*n* = 1
Age of child	
6–12 months	*n* = 7
13–18 months	*n* = 7
Median Age	11 months
Child seight‐for‐length percentile	
Median	95.8%
Number of children at home^a^	
One child	*n* = 6
Two to three children	*n* = 4
More than three children	*n* = 4
Median number of children	Two children

a Some siblings lived in their home country and not in their home in the US.

### 2.2 Study procedures

Semistructured interviews were conducted in the caregiver's language of preference. Most interviews took place at the participant's home, whereas others took place in a clinic conference room or in a neutral location such as a coffee shop. We used a semistructured interview protocol that was developed with input from a paediatrician and from qualitative researchers. The protocol included 22 questions that were intended to elicit the overall experience that parents have with raising a small child, with a focus on key health indicators such as family support and neighbourhood, feeding, play, sleep, and experience with the clinic (see Appendix Table A1). After three initial interviews, there were emerging themes, such as feeding to soothe and justification of weight. To further explore these themes, we added nine questions to the interview protocol for a total of 31 questions. We conducted interviews until thematic saturation was reached and chose to focus on feeding themes for this manuscript. This study was reviewed by an ethics committee and approved by the University of Texas Institutional Review Board; participants signed consent forms before participating and were given assurance of confidentiality.

The research team, comprised of physicians, graduate students, and an undergraduate student, practiced reflexivity by writing memos and engaging in debriefing in order to set aside, as much as possible, any preconceived notions so that the analytic, substantive theory could emerge (Creswell, 2007). Due to all of the participants being patients at the same clinic, the research team reassured the participants that their participation in this qualitative study was voluntary and did not affect any other services or programmes they were receiving at the clinic. The research team also had to manage the possibility of having insider knowledge from previous interactions with the participants; this was done by being intentional about how questions were asked and probed so as to not assume to know anything that the participant meant, especially when using slang or saying “you know.”

### 2.3 Data analysis

The interviews were audio recorded, transcribed verbatim in the language in which they were conducted, and translated with verification from two bilingual researchers. The transcripts were uploaded to NVivo 12 for analysis. Two researchers read through the transcripts independently, following Creswell's analysis process, consisting of a first read‐through of transcripts to identify initial codes, then comparing initial codes between transcripts, expanding these codes, and condensing to final themes (Creswell, 2007). The researchers met periodically to discuss emerging themes, illustrative quotes, and areas of particular interest that appeared repeatedly. The final identified themes were confirmed by all four members of the research team.

### 2.4 Ethical Considerations

This study was approved by the University of Texas Institutional Review Board.

## 3 RESULTS

Interviews took place from December 2018 to February 2019 and lasted 10 to 76 min with a median time of 25 min. A total of 15 interviews were conducted. Eleven of the interviews were in Spanish. The interview of a mother who identified as non‐Hispanic was not included in the analysis, and theme saturation was confirmed without her interview. Demographic data are available in Table 1. Although interview questions covered an array of topics in parenting, several themes emerged regarding feeding practices, which we explore here. There are three major themes: feeding, food choices, and food and weight, which are divided further into subthemes, which can be found in Table 2. All quotes have been given pseudonyms for privacy of participants.

**Table 2:** Descriptions of themes and subthemes

Theme/subtheme	Description
Feeding	
Feeding to appease or soothe, driving overfeeding	Mothers often fed their child in response to crying or to prevent it.
Loose meal structure, feeding when child requests food, especially at night	Mothers did not have a set schedule for feeding their child throughout the day.
Food choices	
Physicians and WIC staff seen as important sources of nutrition information	Mothers often said physicians or WIC staff were the main source of feeding information.
Use of juice	Mothers mostly perceived juice as being unhealthy due to amount of sugar.
Difficulties transitioning to solid food	Mothers expressed not knowing what to feed children when transitioning to solid food.
Food and weight	
Justification and perceptions of weight	Mothers recognized association between type of food/amount of food and child weight. Mothers were mixed in their views of their child's weight status.

Note. WIC = Special Supplemental Nutrition Program for Women, Infants, and Children.

### 3.1 Feeding

#### 3.1.1 Feeding to appease or soothe, driving overfeeding

We found that mothers often fed their child in response to crying or to prevent it. In these situations, they fed their child breastmilk, formula, or cow's milk, depending on the age of the child and options available to the family; about half used both breastmilk and formula while the child was an infant, whereas another half used either breastmilk or bottle almost exclusively. Mothers spoke of feeding milk routinely in preparation for a nap or bedtime as well, with unsuccessful attempts to reduce the quantity of milk. For example, Ana noted, “Sometimes I give him six [ounces], he knows it's not eight, so he'll cry because he knows it's not enough … He knows there's still left. Like it's too soon for the milk to be done”.

Feeding in response to crying or in anticipation of crying generally led to feeding the child large quantities of milk or formula above the American Academy of Pediatrics' recommendations for each developmental stage and for the weight of the child (Hagan, Shaw, & Duncan, 2017). Some mothers such as Mary reflected on their own emotions having a role in overfeeding. “My husband tells me not to give them more. That I'm going to get him into the habit of eating too much. Then he starts to cry and cry. As a mom it hurts me to see them cry. Sometimes my heart wins.” Although some mothers recognized the need to change their habit of feeding in response to crying and opt for scheduled feedings, they feared that the child would cry if they did not receive the milk that they were expecting, and they would have no other way to successfully soothe the child. For example, Alicia recognized that “he drinks too much milk throughout the day, that's not normal. But then again if he didn't get the bottle he would cry”. Interestingly, the foster mother in our study, Nicole, reflected that her child's birth parents “were feeding him whenever he cried. So, they thought that when he cried that that's what he needed, and that's not necessarily what he needed.” Most of the mothers noted the difficulty in breaking a pattern of overfeeding in their children, and several felt stressed, including Elena who noted, “it's really difficult to just be home alone with the babies. You get stressed out, frustrated. It's really complicated.”

#### 3.1.2 Loose meal structure, feeding when the child requests food, especially at night

Most mothers did not have a set schedule for feeding their child; permissive feeding, or feeding in response to the child's cries, was the norm. Although many discussed that they fed the child a set number of meals in a day, the times varied from day to day, and snacks or milk were given at random at the request or cries of the child. A few mothers did have a feeding schedule, but it consisted of a set number of meals that were too frequent for the child's age (Altmann, Hill, Shelov, & Hannemann, 2019). For example, Isabella reported, “Like at nine I give him Gerber [pureed baby food] and then at like ten, at ten I give him food. And then at like twelve yogurt and then at like three, starting—At three I give him food again and like that. Then in the afternoon I give him yogurt again, or soup. Based on if I have any because sometimes it runs out or I don't have any.” Isabella's final comment gives insight to the fact that the sporadic meal times may not be due to choice; the family may be food insecure and only have a certain amount of food for a given day or week, making it difficult to follow a consistent eating schedule.

Most mothers regularly fed their child milk or formula at least once at night, if not two or three times, regardless of the child's age. For example, Alicia reported, “Sometimes he'll sleep through the night, other nights he won't. He'll want to eat a lot… but I don't want him crying throughout the night wanting the bottle and not going back to sleep, so I just let him have it.” However, night‐time feedings are not necessary after 6 months of age as the child is able to safely sleep throughout the night without being fed (Schmitt, 2013). Mary reflected, “My breasts tell me that during the night it's not necessary for him anymore. At his age he should be sleeping the whole night without waking up to eat. But it's very difficult because then he doesn't want to sleep and he wakes up at night because he wants to breastfeed”. Some said that they only breastfed the child for a few minutes, but a significant amount said they gave the child several ounces or entire bottles of milk or formula throughout the night. Rather than commensurately reducing daytime feeding, this significantly added to the amount of milk the child received in a day. Overall, this was a cause of stress and struggle for the mothers as it interrupted their sleep; many felt bad for continuing to give the child a bottle or breast milk but saw no other option because the child cried without it.

### 3.2 Food choices

#### 3.2.1 Physicians and WIC staff seen as important sources of nutrition information

Almost every mother mentioned physician or WIC feeding recommendations spontaneously and in positive regard, even if they were not yet able to implement the changes recommended. The appreciation was evident when Andrea commented, “Because well also at the WIC they tell me what she should already eat or what she should be doing or that in a few months she should be doing this. So it's also helped me to know and like learn.” Speaking with physicians also helped some of the mothers better understand their child's weight status, as Andrea continued, “Because the doctor had talked to me that it's not so much about her physical state, but it's for her health.” Some also mentioned specific nutrition questions that they would ask at future visits. For example, Sofia stated, “I'm going to speak with the doctor to see what they recommend for me to feed him and all of that.” The clinic was seen as a reputable and respectable source of information. One mother noted that living in the US and having access to WIC made it much easier to raise a child and not worry about access to formula or nutritious foods for their child.

#### 3.2.2 Use of juice

Mothers mostly stated they did not give their children large amounts of juice; many mentioned that they were worried about the high sugar content of juice. For example, Ana noted, “It's mostly water because I don't want him getting with a lot of sugar. I don't like sugar.” However, juice was still a part of many of the children's diet. Mothers who did say they gave juice mostly gave the child only small amounts of juice, and some mixed juice with water to reduce the sugar content. This was clear from Andrea's comments: “Yes, but I give her the juices for babies. I add a little bit of water because I think that they're a little sweet… If it was up to her all she would drink is water”. Others that gave juice noted that natural juice or juice that is marketed as “baby” juice is preferable.

#### 3.2.3 Difficulties transitioning to solid food

Across all ages, mothers expressed some difficulty or frustration with transitioning their child to new foods or knowing what to feed them. For some, the content of meals was more distressing; some were bored with what they gave their child and wished they knew something else to give them. For example, Andrea reported, “And what else do I give her? I've already given her soups, I've already given her this. How else can I offer her this vegetable or how else can I offer her this”? Additionally, other than traditional baby foods, such as pureed vegetables or foods traditionally made in the home such as *fideo* soup (noodle soup in a tomato base) or food that the family was eating, mothers struggled to find ways to incorporate a variety into their child's diet. Ana suggested she did not want her child to be picky like her sister's child, but did not know how to be successful: “Yes, I don't to him be a picky eater. Like my nephew is really picky, and that's because my sister will give him more bread and like pasta. And like he doesn't really like vegetables … imagine when he's older… he won't eat any of that.”

For others, the difficulty was timing of meals: how often to feed them in a day, when to start which kinds of foods, and when and how to stop giving a bottle. Most mothers were feeding the child a bottle or milk too many times in a day, holding on to timing schedules from earlier in the child's life. Nicole reported, “Yes, that's difficult, to try to make a baby understand that she shouldn't eat yet, that it's not time,” expressing the difficulty of transitioning infants to solid foods.

Of note, two participants mentioned a difference in the timing of introduction of solid foods into the diet between the United States and Mexico/Central America. They mentioned that in their home countries, they started solid food much earlier around 2 or 3 months of age rather than 6 months, which is a known risk factor for childhood obesity. They did not have a paediatrician for their children in their home country, so having a paediatrician's recommendations on feeding was new to them. This was most clearly expressed by Elisa when she stated, “She tells me: ‘Right now don't give him [solid food] yet because when you go to the paediatrician, they're going to get mad at you.’ I tell her: ‘But, why? In Mexico I …’ And she tells me: ‘It's because here you're not in Mexico. [laughter] It's because raising a child in Mexico is different’.” Adjusting to these new recommendations was a source of some confusion and even guilt or stress for immigrant mothers; one mother was still afraid to start her child on solid foods as a result of this culture change, even though he was 7 months old. Several had trouble because their family is not nearby to provide support; Elena noted that “because well, in my case I've had to struggle on my own, because my family is far away.”

### 3.3 Food and weight

#### 3.3.1 Justification and perceptions of weight

Many mentioned the child's weight when discussing the content of meals or the number of bottles given in a day, making the connection between feeding and weight. Mothers either came to this realization on their own, or were told by their partner or their physician, as evidenced by Mary's reflection: “Well since she was small I had some problems with her weight because since she was about six months she was already eating. She ate a lot. I've also had issues with her because even now she's short. They always tell me at the doctor's that she weighs too much.”

Among those mothers who mentioned their child's weight and diet, only a few spoke of practical steps to take to adjust their feeding routine, whether decreasing the amount of milk being given, or introducing a feeding schedule. For example, Elena shared that “the doctor called me and said that she was a little heavy. But it's because she loves to eat … So, because the doctor told me that she's a little heavy, today I've been organizing… I was thinking that it's better to give her one daily.” Even among these mothers, they noted that the child's behaviour was a barrier to making these changes, as they often cried due to a low tolerance for change in the amount of food or formula given.

Mothers were mixed in their responses to their child's weight status. Most understood that it was an issue of concern, and some minimized or justified their child's weight. Several were not worried about their child's weight, noting that their child was born large and consequently have always wanted to eat more. Among these mothers was Rachel who reflected, “So it's not that I am over‐feeding her, I think that she is just a large child. I give her the recommended portions. It's just that she has that body‐type, so tall, so large.”

Some felt that it was better for the child to be overweight rather than underweight, including Ana: “Honestly, I feel good about it because I know he's eating good. But then I don't want him gaining weight like… because my nephew like I said, he was thinner and because of the same thing he wouldn't really eat good. And I'm glad my baby is not thin.” Some mothers reported their children were underweight or not gaining sufficient weight early in life and compensated for this appropriately by increasing the amount of milk and formula given per physician instructions. However, they had difficulty adjusting back to a normal quantity of milk and formula for the child's age and current weight. A few did not mention weight as an issue.

## 4 DISCUSSION

The Latinx mothers in our study shared important information about how they approach feeding. Overall, their experience is shaped by a strong desire to care and provide for the child. Mothers desire for their child to grow well, and they are generally open to advice and insight from healthcare professionals on healthy feeding practices. At the same time, mothers' desire for the child to be happy and cared for drives permissive feeding styles in which parents respond to or prevent a child's cries or requests by providing food or milk (Horodynski et al., 2018). This phenomenon is well documented and linked to unhealthy weight. Feeding was found to be very emotionally tied; it made mothers sad and frustrated to allow their child to cry instead of feeding them more. Feeding seems largely driven by the child's requests among our mothers interviewed, which is consistent with previous research in Latinx populations (Chaidez & Kaiser, 2011; Davis, Cole, Blake, McKenney‐Shubert, & Peterson, 2016).

Overfeeding at night was especially prominent in our discussions with mothers. This pattern is concerning because mothers consistently feeding a child at night past 6 months of age can lead to overfeeding and also can pose a problem for the child learning to self soothe without food. Feeding with the intention to calm crying predisposes infants to develop a learned response, thus “training” the child to expect a feeding when they cry or are upset (Altmann et al., 2019). Mothers, however, often felt that they had no other option but to continue this style of feeding, continuing a cycle of night‐time feeding.

Although mothers found it difficult to accept feeding advice from healthcare providers in its entirety, some recommendations from healthcare staff did resonate with them. For instance, mothers reported being advised that a diet higher in vegetables and lower in juice and sugar was preferable; however, problems with implementation arose due to child refusal of food or the mother's uncertainty of how to make and provide healthier foods. One can speculate that this is an issue of low food literacy. In addition, the benefits of organic foods may be overemphasized via marketing or messaging to carers, creating a perceived cost barrier to eating healthy foods that does not necessarily need to exist.

As previously observed, other messages from healthcare professionals such as adhering to a feeding schedule and not overfeeding the child milk or formula or introducing solids early were the most difficult for carers to implement (Harrison et al., 2017). It is possible that other life stressors such as living in a new country, financial stressors, lack of social support, as well as culture and family norms may prevent mothers from following through on advice given by healthcare providers (Harrison et al., 2017). A few recent immigrant mothers expressed stress or confusion due to adjusting to feeding recommendations in the US versus their home country, particularly when initiating solid foods, as they had never been given advice on feeding from a physician prior to immigrating to the US. It is important for healthcare providers to ask about norms of feeding in the carer's home country to better understand and address hesitations and questions about a solid food initiation and feeding schedule that promotes healthy weight. (Chaidez, Townsend, & Kaiser, 2011; Harrison et al., 2017)

Overall, most mothers did not appear to change their feeding practices in light of their child's weight status. Although some spoke of plans to change their practices, many had vague plans or no plans to reduce the child's weight; most were happy that their child at least was not underweight. This is consistent with previous studies that have shown that Latinx mothers view excess weight as protecting the child from being underweight (Isasi et al., 2016; Lindsay, Sussner, Greaney, & Peterson, 2011). One can speculate that cultural norms concerning weight and historical fear of not having sufficient access to food contribute to mother's perception of food and weight, although not explicitly expressed.

One possible solution for helping promote healthy feeding habits among this population is offering group clinic visits, during which a facilitator and physician could spend much more time with a group than they could with individual patients, relaying important feeding and nutrition guidance for each developmental stage. Most mothers did not have a robust support system for raising their child; many had family living in different countries or not available to help, and most worked or stayed at home where they were mostly isolated from social networks. Placing the parent in a cohort of other new parents with children of the same age could help to build community and a space to share ideas, frustrations, and successes, helping parents make practical steps toward behavioural change (Bloomfield & Rising, 2013).

Motivational interviewing may also help mothers to identify their own priorities and reasons to make improvements to their child's diet. This style helps to align the healthcare provider with the parent as an ally because both are invested in the child's well‐being, opening communication and allowing the mother to set goals that are reasonable for her and her child (Borrelli, Tooley, & Scott‐Sheldon, 2015).

### 4.1 Strengths and limitations

Our study was small and only focused on mothers at one clinic in Central Texas; we recruited at the clinic, which may have captured a more motivated or resourced group of mothers. This means that our findings may not be generalizable to all Latinx mothers of young children. We collected self‐reported data, which can be affected by social response bias. However, our study had several strengths as well. Our sample included mothers with children at various stages of development and eating practices (e.g., some children were walking or eating solid foods vs. others who were not). Additionally, the data showed that the mothers represented at least four different Latin American countries (e.g., Mexico and El Salvador). Lastly, because these families attend the same clinic, we may be able to conduct follow‐up studies with the same families to understand changes over time.

## 5 CONCLUSIONS

Our study contributes to the existing literature by advancing the understanding of feeding practices and experiences of Latinx mothers with young children at risk for childhood obesity. Mothers' desire to provide for their child and prevent their child from crying can drive permissive feeding practices that are counter to maintaining a healthy weight. There are opportunities to intervene to potentially prevent obesogenic feeding habits by coaching mothers on culturally appropriate alternative soothing techniques, including swaddling, singing, and bathing, appropriate feeding timing and portions, and on healthy weight. Although mothers value the opinion of healthcare professionals, they have difficulty implementing their recommendations. Alternative care models and methods of communication including group clinic visits and motivational interviewing may be better strategies for helping mothers implement recommendations. Further research is also needed so providers can better understand and address the effects of cultural norms, food insecurity, and sleep and routines on feeding practices and future childhood obesity.

## CONFLICTS OF INTEREST

The authors declare that they have no conflicts of interest.

## CONTRIBUTIONS

HM and GDT recruited participants. HM and MG designed the study. EAJ provided input on the design and execution of the study as an expert in qualitative research. HM, CV, and GDT conducted interviews. HM transcribed English interviews. GDT translated Spanish interviews with input from MG and CV. HM and CV analysed the data. HM, CV, and GDT wrote the paper. EAJ and MG provided input on the paper.

## Appendix A.

**Table A1:** Semistructured interview protocol

Topic: Parenting experience	What has your experience been like parenting an infant/toddler?
	Which things are most difficult?
	What has been the most rewarding?
	Who or what helps you?
Topic: Home	Could you tell me about where you live?‡
	Who lives in the house?‡
	Are you satisfied with the amount of support you receive from those around you?‡
	Do you feel safe where you live?‡
	What do you like about where you live? Dislike?‡
Topic: Feeding	Could you tell me about mealtimes and snacks with your child?
	How do you decide what to feed your child?
	What do you find challenging about feeding your child?
	What does your child do during mealtimes?
	What kinds of beverages does your child drink?
Topic: Sleep	Could you talk about what bedtime is like for your child?
	Where does your child sleep?
	How do you soothe your child?
	For how long does your child sleep?
	Does your child nap?
Topic: Play and TV	Could you tell me about what free time and playtime look like for your child?
	Do they have favourite activities or toys?
	Do they use a phone or iPad or watch TV?‡
Topic: Healthy Habits	What behaviours or actions do you see as healthy for your child?
	Who or what helps you to make healthy choices for your child?
Topic: Weight	Has your doctor talked to you about your child's weight?‡
	How do you feel about it?‡
	What do your friends and family say or think?‡
Topic: Clinic	Could you tell me about how your experience has been like with this clinic?
	How has it been helpful? Hurtful?
	What other support services would you like to have?
Topic: Other	Is there anything else I missed that you think is important?

‡ These questions were added after analysis of the first three interviews.

## References

[mcn12983-bib-0001] Altmann, T. R. , Hill, D. L. , Shelov, S. P. , & Hannemann, R. E. (2019). Caring for your baby and young child. New York: Bantam Books.

[mcn12983-bib-0002] Azad, M. B. , Vehling, L. , Chan, D. , Klopp, A. , Nickel, N. C. , McGavock, J. M. , … on behalf of the CHILD Study Investigators (2018). Infant feeding and weight gain: Separating breast milk from breastfeeding and formula from food. Pediatrics, 142(4), e20181092 10.1542/peds.2018-1092 30249624

[mcn12983-bib-0003] Bloomfield, J. , & Rising, S. S. (2013). CenteringParenting: An innovative dyad model for group mother‐infant care. Journal of Midwifery and Women's Health, 58(6), 683–689. 10.1111/jmwh.12132 24406037

[mcn12983-bib-0004] Borrelli, B. , Tooley, E. M. , & Scott‐Sheldon, L. A. (2015). Motivational interviewing for parent‐child health interventions: A systematic review and meta‐analysis. Pediatric Dentistry, 37(3), 254–265. 26063554

[mcn12983-bib-0005] Chaidez, V. , & Kaiser, L. L. (2011). Validation of an instrument to assess toddler feeding practices of Latino mothers. Appetite, 57(1), 229–236. 10.1016/j.appet.2011.05.106 21600943

[mcn12983-bib-0006] Chaidez, V. , Townsend, M. , & Kaiser, L. L. (2011). Toddler‐feeding practices among Mexican American mothers. A qualitative study. Appetite, 56(3), 629–632. 10.1016/j.appet.2011.02.015 21354235

[mcn12983-bib-0008] Creswell, J. W. (2007). Qualitative inquiry & research design: Choosing among five approaches. Thousand Oaks: Sage Publications.

[mcn12983-bib-0009] Davis, R. E. , Cole, S. M. , Blake, C. E. , McKenney‐Shubert, S. J. , & Peterson, K. E. (2016). Eat, play, view, sleep: Exploring Mexican American mothers' perceptions of decision making for four behaviors associated with childhood obesity risk. Appetite, 101, 104–113. 10.1016/j.appet.2016.02.158 26944228 PMC5533082

[mcn12983-bib-0010] Gaffney, K. F. , Brito, A. V. , Kitsantas, P. , Kermer, D. A. , Pereddo, G. , & Ramos, K. M. (2018). Institute of medicine early infant feeding recommendations for childhood obesity prevention: Implementation by immigrant mothers from Central America. Journal of Pediatric Nursing, 40, 27–33. 10.1016/j.pedn.2018.02.017 29776476 PMC5962027

[mcn12983-bib-0011] Geserick, M. , Vogel, M. , Gausche, R. , Lipek, T. , Spielau, U. , Keller, E. , … Körner, A. (2018). Acceleration of BMI in early childhood and risk of sustained obesity. The New England Journal of Medicine, 379(14), 1303–1312. 10.1056/NEJMoa1803527 30281992

[mcn12983-bib-0012] Gross, R. , Mendelsohn, A. , Fierman, A. , Racine, A. , & Messito, M. (2012). Food insecurity and obesogenic maternal infant feeding styles and practices in low‐income families. Pediatrics, 130(2), 254–261. 10.1542/peds.2011-3588 22826569

[mcn12983-bib-0013] Hagan, J. F. , Shaw, J. S. , & Duncan, P. M. (2017). Bright futures: Guidelines for health supervision of infants, children, and adolescents. Elk Grove Village: American Academy of Pediatrics.

[mcn12983-bib-0014] Hales, C. M. , Fryar, C. D. , Carroll, M. D. , Freedman, D. S. , & Ogden, C. L. (2018). Trends in obesity and severe obesity prevalence in US youth and adults by sex and age, 2007‐2008 to 2015‐2016. JAMA, 319, 1723–1725. 10.1001/jama.2018.3060 29570750 PMC5876828

[mcn12983-bib-0015] Harrison, M. , Brodribb, W. , & Hepworth, J. (2017). A qualitative systematic review of maternal infant feeding practices in transitioning from milk feeds to family foods. Maternal & Child Nutrition, 13(2). 10.1111/mcn.12360 PMC6865989 27696658

[mcn12983-bib-0016] Helle, C. , Hillesund, E. R. , Wills, A. K. , & Øverby, N. C. (2019). Evaluation of an eHealth intervention aiming to promote healthy food habits from infancy ‐the Norwegian randomized controlled trial Early Food for Future Health. The International Journal of Behavioral Nutrition and Physical Activity, 16(1), 1–16. 10.1186/s12966-018-0763-4 30606197 PMC6318886

[mcn12983-bib-0017] Horodynski, M. A. , Brophy‐Herb, H. E. , Martoccio, T. L. , Contreras, D. , Peterson, K. , Shattuck, M. , … Lumeng, J. C. (2018). Familial psychosocial risk classes and preschooler body mass index: The moderating effect of caregiver feeding style. Appetite, 123, 216–224. 10.1016/j.appet.2017.12.025 29287633

[mcn12983-bib-0018] Isasi, C. , Rastogi, D. , & Molina, K. (2016). Health issues in Hispanic/Latino youth. Journal of Latina/o Psychology, 4(2), 67–82. 10.1037/lat0000054 27347457 PMC4915390

[mcn12983-bib-0020] Li, R. , Scanlon, K. S. , May, A. , Rose, C. , & Birch, L. (2014). Bottle‐feeding practices during early infancy and eating behaviors at 6 years of age. Pediatrics, 134(Suppl 1(Supplement)), S70–S77. 10.1542/peds.2014-0646L 25183759 PMC4258847

[mcn12983-bib-0021] Lindsay, A. C. , Sussner, K. M. , Greaney, M. L. , & Peterson, K. E. (2011). Latina mothers' beliefs and practices related to weight status, feeding, and the development of child overweight. Public Health Nursing, 28(2), 107–118. 10.1111/j.1525-1446.2010.00906.x 21442018 PMC3063070

[mcn12983-bib-0022] McGeorge, K. , Milne, L. , Cotton, L. , & Whelan, T. (2015). Effects of infant and maternal sensory processing on infant fussing, crying, and sleep. Infant Mental Health Journal, 36(3), 275–286. 10.1002/imhj.21510 25892527

[mcn12983-bib-0023] Nemiary, D. , Shim, R. , Mattox, G. , & Holden, K. (2012). The relationship between obesity and depression among adolescents. Psychiatric Annals, 42, 305–308. 10.3928/00485713-20120806-09 23976799 PMC3749079

[mcn12983-bib-0025] Ogden, C. L. , Carroll, M. D. , Lawman, H. G. , Fryar, C. D. , Kruszon‐Moran, D. , Kit, B. K. , & Flegal, K. M. (2016). Trends in obesity prevalence among children and adolescents in the United States, 1988‐1994 through 2013‐2014. JAMA, 315(21), 2292–2299. 10.1001/jama.2016.6361 27272581 PMC6361521

[mcn12983-bib-0026] Pan, L. , Freedman, D. S. , Sharma, A. J. , Castellanos‐Brown, K. , Park, S. , Smith, R. B. , & Blanck, H. M. (2016). Trends in obesity among participants aged 2–4 years in the Special Supplemental Nutrition Program for Women, Infants, and Children—United States, 2000–2014. Morbidity and Mortality Weekly Report (MMWR), 65, 1256–1260. 10.15585/mmwr.mm6545a2 27855143

[mcn12983-bib-0028] Schmitt, B. D. (2013). Your Child's Health: The Parents’ One‐stop Reference Guide to: Symptoms, Emergencies, Common Illnesses, Behavior Problems, Healthy Development. New York: Bantam Books.

[mcn12983-bib-0030] Shloim, N. , Edelson, L. , Martin, N. , & Hetherington, M. (2015). Parenting styles, feeding styles, feeding practices, and weight status in 4–12 year old children: A systematic review of the literature. Frontiers in Psychology, 6, 1849 10.3389/fpsyg.2015.01849 26696920 PMC4677105

[mcn12983-bib-0032] Weng, S. F. , Redsell, S. A. , Swift, J. A. , Yang, M. , & Glazebrook, C. P. (2012). Systematic review and meta‐analyses of risk factors for childhood overweight identifiable during infancy. Archives of Disease in Childhood, 97(12), 1019–1026. 10.1136/archdischild-2012-302263 23109090 PMC3512440

[mcn12983-bib-0033] Woo Baidal, J. A. , Locks, L. M. , Cheng, E. R. , Blake‐Lamb, T. L. , Perkins, M. E. , & Taveras, E. M. (2016). Risk factors for childhood obesity in the first 1,000 days: A systematic review. American Journal of Preventive Medicine, 50, 761–779. 10.1016/j.amepre.2015.11.012 26916261

